# Engaging the private sector as part of HIV pre-exposure prophylaxis service delivery in Vietnam: a comparative analysis of uptake, persistence and HIV seroconversion from 2018 to 2023

**DOI:** 10.3389/frph.2024.1439461

**Published:** 2025-01-21

**Authors:** Bao Ngoc Vu, Kimberly Green, Huong Phan, Long Tran, Phuong Phan, Tham Tran, Linh Doan, Yen Vu, Chau Pham, Dao Nguyen, Anh Doan, Trang Ngo, Phuong Tran, Vuong Nguyen, Bieu Nguyen, Thai Phan, Ha Nguyen

**Affiliations:** ^1^ Southeast Asia Hub, PATH, Hanoi, Vietnam; ^2^ Ministry of Health, Vietnam Administration of HIV/AIDS Control, Hanoi, Vietnam; ^3^ Office of Health, United States Agency for International Development (Vietnam), Hanoi, Vietnam; ^4^ Southeast Asia Hub, PATH, Ho Chi Minh City, Vietnam

**Keywords:** HIV prevention, pre-exposure prophylaxis, private sector engagement, key populations, Vietnam

## Abstract

**Background:**

In Vietnam, PrEP was introduced in 2017 and scaled up from 2019. Private sector engagement (PSE) in PrEP service delivery was deployed as a strategy from the start to increase PrEP access. We assessed the effectiveness of this approach to inform ongoing efforts to accelerate epidemic control by 2030.

**Methods:**

We implemented a process evaluation with longitudinal design using retrospective programmatic data collected and uploaded onto a secure online system (HMED) from 23 public and 17 private PrEP clinics in Hanoi, Ho Chi Minh City (HCMC), and Dong Nai. We measured the effectiveness of PrEP service delivery by PrEP initiation/reinitiation, uptake, persistence, discontinuation, and HIV seroconversion. We used the Kaplan-Meier time-to-event approach to estimate PrEP persistence and mixed-effects logistic regression analysis to assess factors associated with the PrEP persistence.

**Results:**

From October 2017 to September 2023, 29,944 individuals initiated PrEP, and among these, 79.3% started PrEP at a private sector clinic while 20.7% initiated in a public sector clinic. The median duration of PrEP use persistence at private clinics was significantly longer than that at public clinics (268 days vs. 148 days, respectively). Mixed-effects logistic regression analysis results indicated a significant statistical association between PrEP persistence for at least three months and initiating PrEP at a private clinic [adjusted odds ratio [aOR] = 4.28; 95% confidence interval [CI]: 2.96–6.19], opting for TelePrEP (aOR = 3.42; 95% CI: 2.12–5.53), or being 20 years of age or older (aOR = 1.86; 95% CI: 1.62–2.13). HIV seroconversion was significantly lower among PrEP users at private clinics compared to public clinics (0.03 vs. 0.13 per 100 person-years, respectively; *p* < 0.01).

**Conclusion:**

Offering choice in PrEP service delivery options is essential to increase access and uptake. Private-sector PrEP providers play a pivotal role in increasing PrEP uptake and coverage in Vietnam, and will be critical to delivery of new long-acting options.

## 1 Introduction

PrEP is a high impact HIV prevention tool that could drive HIV transmission toward elimination if scaled up to achieve high coverage (1). Although oral pre-exposure prophylaxis (PrEP) has been proven to be highly effective in preventing HIV infection, global PrEP uptake has been inadequate to effectively accelerate progress towards HIV epidemic control in many parts of the world (1, 2). While the total number of people using PrEP globally has risen from over 233 000 in 2019 to over 3.5 million in 2023, large-scale PrEP coverage remains limited to a small number of countries (3). This represents only 35% of the global PrEP target of ten million set for 2025 (3). According to the UNAIDS Global AIDS Monitoring 2023, the largest gap in PrEP uptake is in Asia-Pacific, where nearly a quarter of new HIV infections occurred in 2022 (3). Scaling up PrEP uptake requires innovations in differentiated, simplified, demedicalized and integrated models of care, along with enabling service convenience, choice and confidentiality to ensure that all those who need PrEP can access it (4).

Leveraging the private sector as part of HIV service delivery can enhance person-centered care, diversify funding streams, fosters innovation, and improve the efficiency and sustainability of the HIV response (5, 6). Private sector health care providers may include private hospitals and clinics, pharmacies, drug shops, social-enterprises, community-based organizations, and onsite, mobile and telehealth services. While the World Health Organization (WHO), the President's Emergency Plan for AIDS Relief (PEPFAR), the Global Fund (GF), the United States Agency for International Development (USAID), country leaders and others have called for greater PSE to optimize HIV service choice and sustainability, overall access to private sector HIV services remains limited in many settings (5, 7–9).

As part of efforts to increase service options and domestic financing, low and middle income countries (LMIC) are increasingly engaging private sector service providers, including private clinics and pharmacies, in the delivery of PrEP services through either pure commercial, partial subsidy/mixed financing models or free service mechanisms. Studies across Asia-Pacific (i.e., Australia, New Zealand, India, Philippines, Taiwan, Thailand and Vietnam) and USA and Africa (i.e., South Africa and Kenya) indicate that private sector PrEP services are acceptable in the settings where they've been offered, and that private service providers are capable of safely starting and managing clients on PrEP, reaching more individuals typically underserved by public-sector clinics and increasing overall PrEP coverage (5, 10). Despite this PrEP is commonly delivered at public sector health facilities, while private sector PrEP service delivery is often limited to small scale pilots. There are also concerns about the uneven quality and affordability of private sector health services (11, 12). In all, little is known about PrEP service delivery at private sector health facilities.

Recognizing the importance of PrEP, the government of Vietnam adopted the WHO recommendation to implement oral PrEP as one of the key interventions in the National Strategy for Ending AIDS by 2030 (13, 14)^1^. PrEP was introduced in Vietnam in 2017 through the Prepped for PrEP (P4P) pilot, and the Ministry of Health (MOH) approved and implemented two national PrEP scale-up plans for the period 2018–2020 and 2021–2025, with the goal of achieving PrEP coverage for 30% of total MSM by 2025 (15, 16)^1^. These national PrEP scale-up plans, funded by PEPFAR and the GF, have been supported by international development organizations (WHO, USAID, and the US CDC) and global health organizations, such as PATH and FHI 360. Vietnam is a leading country in Southeast Asia, after Thailand, in implementing a policy and national program for PrEP soon after the WHO recommendation in 2015. As of 2023, 67,191 people have received PrEP at least once in the last 12 months ranking Vietnam first in PrEP uptake in the Asia-Pacific region significantly ahead of Australia and Thailand (3).

Learning from the P4P pilot (4), the Vietnam Administration of HIV/AIDS Control (VAAC), which is tasked by the Ministry of Health to lead the national PrEP scale-up plan, encouraged provinces and cities to engage private sector service providers in PrEP implementation and put in place guidelines to simplify PrEP delivery in private sector clinics. To date, 219 clinics (170 public and 49 private) offer PrEP services across 29 out of 63 provinces in the country. Notably, private clinics, which accounted for just one-fifth (22.3%) of total PrEP clinics, served nearly half (48%) of total PrEP clients in the country. However, the scale and scope of PSE in PrEP service delivery varies across provinces and cities in Vietnam.

This paper aims to generate evidence on the PSE approach to PrEP service delivery in a real-world setting. In this paper, we present findings from an analysis of routine programmatic data to assess the effectiveness of private sector engagement in PrEP service delivery, implemented in three provinces (Hanoi, Ho Chi Minh City, and Dong Nai) and supported by PEPFAR through the USAID/PATH Healthy Markets and USAID/PATH Support for Technical Excellence and Private Sector Sustainability in Vietnam (STEPS) projects.

## 2 Materials and methods

### 2.1 Study design and data source

We conducted an analysis of routine programmatic data among PrEP users enrolled in forty clinics (23 public and 17 private sector) in three provinces (Hanoi, Ho Chi Minh City, and Dong Nai) to assess the effectiveness of private sector engagement in PrEP service delivery compared to public sector PrEP service delivery. The forty sites included in the analysis represent all clinics supported by PEPFAR through USAID/PATH Healthy Markets and USAID/PATH STEPS projects from 01 October 2018 to 30 September 2023 in the three above mentioned provinces. Routine M&E data from all clients enrolled on PrEP were included in the analysis. Data available included client enrollment, continuation/persistence on PrEP, and HIV sero-conversion. We extracted data from aggregated service monitoring reports at the following time periods: PrEP initiation (T0) and every follow-up visit at month one (M1), three months (M3), six months (M6), nine months (M9) and 12 months (M12). In this analysis, we defined the effectiveness of PrEP service delivery as the ability to enroll and maintain people on PrEP, and to prevent HIV acquisition (17). Respective indicators for measurement of PrEP service delivery effectiveness were: (1) PrEP uptake; (2) PrEP persistence rate at month 3 (M3) and ongoing persistence; and (3) HIV seroconversion rate at the month one and at follow-up visits.

### 2.2 Description of the PrEP service delivery at public and private clinics

The PrEP program was implemented in three provinces (Hanoi, Ho Chi Minh City, and Dong Nai), which were purposively selected based on high HIV burden, the largest number of key populations (KP), such as men who have sex with men (MSM), people who inject drugs (PWID), female sex workers (FSW), and transgender women (TGW), and the United States President's Emergency Plan for AIDS Relief (PEPFAR) epidemic control investments in these locations. We engaged a total of 40 clinics, including 23 public and 17 private clinics in delivery of PrEP services. PrEP at public clinics was delivered at the outpatient clinics located at district health centers which were originally established to provide antiretroviral therapy (ART), while PrEP at private clinics was offered at KP-led or KP friendly clinics as part of a primary health care (PHC) one-stop shop (OSS) approach providing person-centered care services, including HIV testing, post-exposure prophylaxis (PEP), sexually transmitted infection (STI) testing and treatment, ART, hepatitis B and C testing and treatment, noncommunicable disease management, mental health services and/or transgender-affirming care. Public clinics provided on-site PrEP services serving clients who were referred to or linked by key population community-based organizations (KP CBOs) and HIV counseling and testing (HCT) centers or were walk-in clients. Taking advantage of having their own network of peer educators or a partnership with KP CBOs, four out of 17 private clinics offered mobile and/or TelePrEP in addition to on-site services for clients living far away or unable to visit the clinic for PrEP initiation and/or follow-up. Free oral PrEP services were provided at all public and private clinics, while private clinics also offered a fee-based PrEP option. KP CBOs played a critical role in providing information about PrEP to clients who tested HIV negative, conducting a pre-screen for eligibility and then referring these clients for PrEP services at public or private clinics. In addition, because most KP CBOs are heavily engaged online, they posted information on PrEP and where to go for PrEP services on their social media and websites. PrEP services were consistently advertised online through two separate dedicated Facebook pages—Rainbow Village reaching MSM and Be Me Be Happy reaching TGW. These Facebook pages included online peer influencers who screened for HIV testing and PrEP needs and made direct referrals to the KP-led clinics. An online HIV self-testing and PrEP self-screener also supported client awareness of and linkages to PrEP.

Clinic staff, including clinicians, pharmacists, counselors, and lab technicians, received a three-day training to provide daily oral PrEP and on-demand PrEP following the national guidelines on HIV care and treatment.^2^ Based on the national guidelines, the PrEP program dispensed oral tenofovir disoproxil fumarate and emtricitabine (TDF/FTC 300 mg/200 mg) as a fixed-dose combination and needed a clinical follow-up visit one month after PrEP initiation and then every three months after that. PrEP prescription refills were scheduled at 1-month, 3-month, 6-month, 9-month, and 12-month clinical visits after initiation as other necessary indicators (HIV test results, risk assessment, and prescription renewal) were done at these appointments.

PrEP was indicated for specific populations at substantial risk of HIV, including MSM, serodiscordant couples (SDC) where the partner with HIV is not virally suppressed, TGW, PWID, FSW, individuals with a recent history of STIs, those who have undergone postexposure prophylaxis (PEP), individuals who recognize their own risk and request PrEP. Before initiating PrEP, clients were screened for eligibility through a standard risk assessment form testing for HIV (using antibody/antigen fourth-generation tests for as recommended by the national guidelines), creatinine, hepatitis B and C virus, syphilis, and chlamydia and gonorrhea (if available), and a clinical exam. If eligible, clients were prescribed same day PrEP drugs for one month and scheduled revisits every three months for PrEP refills, HIV testing, and/or creatinine. According to the national guidelines, clients who want to stop PrEP (e.g., when they report no longer being at risk of HIV infection or preferred another HIV prevention method) were recommended to have a consultation with their healthcare provider to retest for HIV, reassess risk, receive any counseling as needed, and recording the reason for stopping PrEP in the client medical records.

### 2.3 Data capture, measures, and analysis

Clinics captured individual-based data on real-time PrEP service utilization using electronic medical records (EMR) uploaded onto a secure online system of the national PrEP program (HMED PrEP). The HMED system facilitates the capture of comprehensive clinical and demographic data, such as age, sex, population group, start date for PrEP, HIV test result, PrEP regimen, prescription, and adherence at each scheduled visit, date of stopping PrEP, the reason for stopping, and referral source. Health care providers directly imported data at the clinics to ensure contemporaneous and accurate documentation of client interactions. Each clinic had an HMED account to import data on a daily or weekly basis and export data for monthly/quarterly progress indicator reports required by the government and the donor. To safeguard the quality and integrity of the data, Monitoring, Evaluation and Learning (MEL) staff at PATH, the clinics, CBOs and local health authorities employed Data Quality Audit (DQA) and Continuous Quality Improvement (CQI) procedures. The DQA process involved routine validation checks to identify and correct inconsistencies or missing data for enhancing data completeness and accuracy. Concurrently, the CQI visits to PrEP clinics were conducted to identify real-time data gaps, provide feedback, and support for improvement of data quality at the clinics. These quality control measures aimed at enhancing the reliability and validity of the dataset and ensuring robust and accurate data analysis.

The routine programmatic data on PrEP service delivery from the 40 public and private clinics was de-identified, synthesized, verified, and transferred to Stata 14 analysis. As part of routine monitoring, these PrEP clinics measured and reported core indicators required by the national PrEP program, including PrEP initiation, PrEP uptake, PrEP continuation at three months, and PrEP discontinuation including HIV seroconversion. In this study key variables were operationally defined as follows:

- PrEP initiation was defined as the number of individuals newly enrolled on oral PrEP to prevent HIV infection, reported quarterly.
- PrEP uptake was defined as the number of people who received oral PrEP at least once within the last 12 months.
- PrEP continuation was defined as receiving a PrEP drug refill at 1, 2, 3, 6, 9, and 12 months after PrEP initiation.
- PrEP discontinuation was defined as formally stopping PrEP use or having no show visit at 1,2,3,6,9, and 12 months after PrEP initiation and never returning to the clinic within 30 days.
- Time on PrEP was calculated as the number of days between starting PrEP and stopping PrEP or being lost to follow-up.
- PrEP re-initiation was identified as a visit where a participant reported currently taking PrEP after having previously indicated discontinuation.
- HIV seroconversion was defined as HIV infection detected during the follow-up visit in a participant who was HIV-seronegative at the time of enrollment.
- HIV infection was defined as positive HIV diagnosis determined by the national HIV testing algorithm, using three different rapid diagnostic tests (RDTs) or the gold standard enzyme immunoassay (EIA) or enzyme-linked immunosorbent assay (ELISA).

The main outcome of interest was PrEP persistence or continuation over time, as well as the factors associated with PrEP persistence between private and public sector PrEP service delivery models. PrEP persistence referred to the length of time that a person continued to refill PrEP prescriptions without an interruption of more than 30 days. PrEP persistence rate was defined as the proportion of participants initiating PrEP within 30 days of enrollment on PrEP at the three months, six months, nine months, or 12 months assessment regardless of any time off PrEP in the interim retention. Clinics monitored PrEP prescription refills from a person's first PrEP prescription date until there was a gap of more than 30 days between refills or a complete discontinuation of PrEP. We calculated the end date in PrEP persistence as the date the prescribed PrEP drugs would run out (daily PrEP). If the person did not refill a PrEP prescription within 30 days of the end date, we considered the person non-persistent on the 31st day. We used a 30-day gap to define non-persistence because a longer gap might result in drug levels too low to confer protection.

Descriptive analysis was applied to describe the PrEP initiation and reinitiation, PrEP uptake, PrEP discontinuation, and the reasons for discontinuation after enrollment at public and private clinics. The statistical analysis, using the Chi-square, Fisher exact test, or *T*-test was employed to compares the difference in outcomes by factors. To examine factors associated with PrEP persistence for at least 3 months, a mixed-effects logistic regression was applied to control for individual-level repeated measurements. The primary outcome variable was PrEP persistence for at least 3 months. Independent variables included demographic factors (age group, KP type), type of PrEP service provider, PrEP service delivery model, and use of fee-based PrEP services. A random factor at the individual level was included in the model to account for repeated measurements, along with the time variable to test for interactions. Odds ratios (OR) with 95% confidence intervals (95% CI) were reported, and a significant level of *p*-value of 0.05 was applied to all statistical analyses.

We used Kaplan-Meier survival analysis to calculate PrEP persistence because the enrolment of PrEP clients was rolling over time. The Kaplan-Meier approach is one of the best options to measure the fraction of subjects living for a certain amount of time after treatment (18, 19). We utilized the Kaplan-Meier time-to-event method to estimate the duration of persistence in months and calculated the percentage of PrEP persistence at different time points after PrEP initiation (i.e., 1, 3, 6, 9, 12, 18, and 24 months). The probabilities derived from each interval were used to calculate the overall likelihood of the continuation occurring at different time points. Kaplan-Meier survival curves were generated to identify the differences of PrEP persistence by type of PrEP service providers (private vs. public clinics). This study followed clients overtime at scheduled visits from PrEP initiation (M0) to 12-month visits (M12).

HIV seroconversion rate (or HIV incidence rate) among PrEP users was defined as the number of HIV infections per 100 person-years (PY) of follow up after PrEP initiation. The date of infection was estimated as the midpoint between the date of the last negative screening test and the date of the first positive one. Follow-up time was calculated from PrEP initiation to HIV infection or the earlier participant's HIV-negative screening test. Where HIV seroconversions occurred among clients taking PrEP, a case report was prepared detailing when the HIV seroconversion took place, probable cause, and ART status.

## 3 Results

### 3.1 PrEP initiation

**Figure 1:**
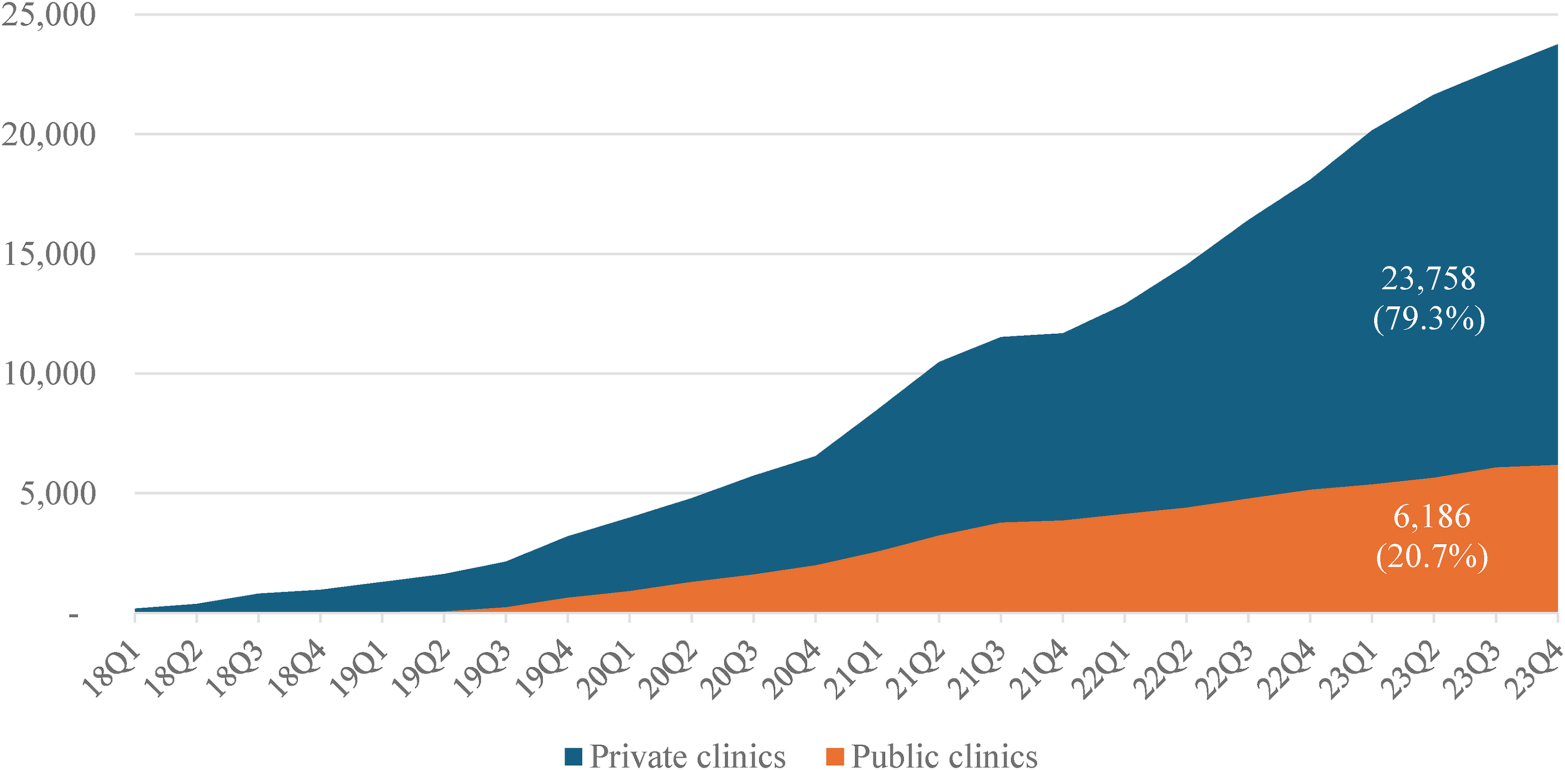
Cumulative new PrEP users enrolled quarterly (Q) by public and private clinics, 2018–2023.

From 01 October 2017 to 30 September 2023, 40 clinics (17 private and 23 public) initiated PrEP for a total of 29,944 clients, of which private clinics contributed four times higher initiations than public clinics (79.3% vs. 20.7%, respectively), and the difference in the number of PrEP initiations at private and public clinics on a quarterly (Q) basis was statistically different (Chi-square test, *p* < 0.001) (Figure 1).

Table 1 shows characteristics of PrEP clients at public and private clinics. PrEP clients at private clinics were significantly younger than that at public clinics (median age: 25 vs. 27, respectively; *p* < 0.001). Overall, the vast majority of PrEP clients at either private or public clinics were at 20–49 years (95% and 94%, respectively). The proportion of clients at 15–19 years at private clinics was slightly higher than that at public clinics (3.7% vs. 2.7%, respectively), while the proportion of clients at 50 years and older at public clinics was significantly higher than that at private clinics (3.4% vs. 1.3%, respectively; *p* < 0.001). The proportion of male clients at private clinics was significantly higher than that at public clinics (94.7% vs. 80.4%, respectively), while the proportion of female clients at public clinics was significantly higher than that at private clinics (19.6% vs. 5.3%, respectively; *p* < 0.001). Similarly, the proportion of MSM clients at private clinics was significantly higher than that at public clinics (89.3% vs. 66.7%, respectively). Notably, the proportion of TGW clients at private clinics was significantly higher than that at public clinics (4% vs. 0.5%, respectively), while the proportion of SDC, PWID and FSW clients at public clinics was significantly higher than that at private clinics (21.9%, 3% and 4.3% vs. 3.7%, 0.1% and 1.8%, respectively; *p* < 0.001). PrEP clients at private clinics accounted for two-thirds (69.7%) of total clients in HCMC, while those only accounted for 11.5% or 18.8% in Dong Nai and Hanoi.

**Table 1:** Characteristics of clients who started PrEP at public and private clinics.

Characteristic	Private clinics	Public clinics	Total	*p* value
	*n* = 23,758	*n* = 6,186	*n* = 29,944	
Age in years, median age	25	27	26	<0.001***^,^^a^
15–19	883 (3.7%)	165 (2.7%)	1,048 (3.5%)	<0.001***^,^^b^
20–24	6,572 (27.7%)	1,658 (26.8%)	8,230 (27.5%)	
25–49	15,990 (67.3%)	4,155 (67.2%)	20,145 (67.3%)	
50+	313 (1.3%)	208 (3.4%)	521 (1.7%)	
Sex				
Male	22,506 (94.7%)	4,976 (80.4%)	27,482 (91.8%)	<0.001***^,^^b^
Female	1,252 (5.3%)	1,210 (19.6%)	2,462 (8.2%)	
Key population group				
MSM	21,227 (89.3%)	4,125 (66.7%)	25,352 (84.7%)	<0.001***^,^^b^
TGW	959 (4%)	30 (0.5%)	989 (3.3%)	
PWID	35 (0.1%)	184 (3%)	219 (0.7%)	
FSW	418 (1.8%)	266 (4.3%)	684 (2.3%)	
SDC	885 (3.7%)	1,355 (21.9%)	2,240 (7.5%)	
Others	234 (1%)	226 (3.7%)	460 (1.5%)	
Residence				
Hanoi	4,462 (18.8%)	1,595 (25.8%)	6,057 (20.2%)	<0.001***^,^^b^
Ho Chi Minh City	16,552 (69.7%)	1,953 (31.6%)	18,505 (61.8%)	
Dong Nai	2,744 (11.5%)	2,638 (42.6%)	5,382 (18%)	
Year of PrEP initiation				
2018	965 (4.1%)	41 (0.7%)	1,006 (3.4%)	<0.001***^,^^b^
2019	2,239 (9.4%)	592 (9.6%)	2,831 (9.5%)	
2020	3,342 (14.1%)	1,353 (21.9%)	4,695 (15.7%)	
2021	5,143 (21.6%)	1,873 (30.3%)	7,016 (23.4%)	
2022	6,411 (27.0%)	1,290 (20.9%)	7,701 (25.7%)	
2023	5,658 (23.8%)	1,037 (16.8%)	6,695 (22.4%)	

^a^ *T*-test.

^b^ Chi square test.

*** *p* < 0.001.

### 3.2 PrEP uptake

**Figure 2:**
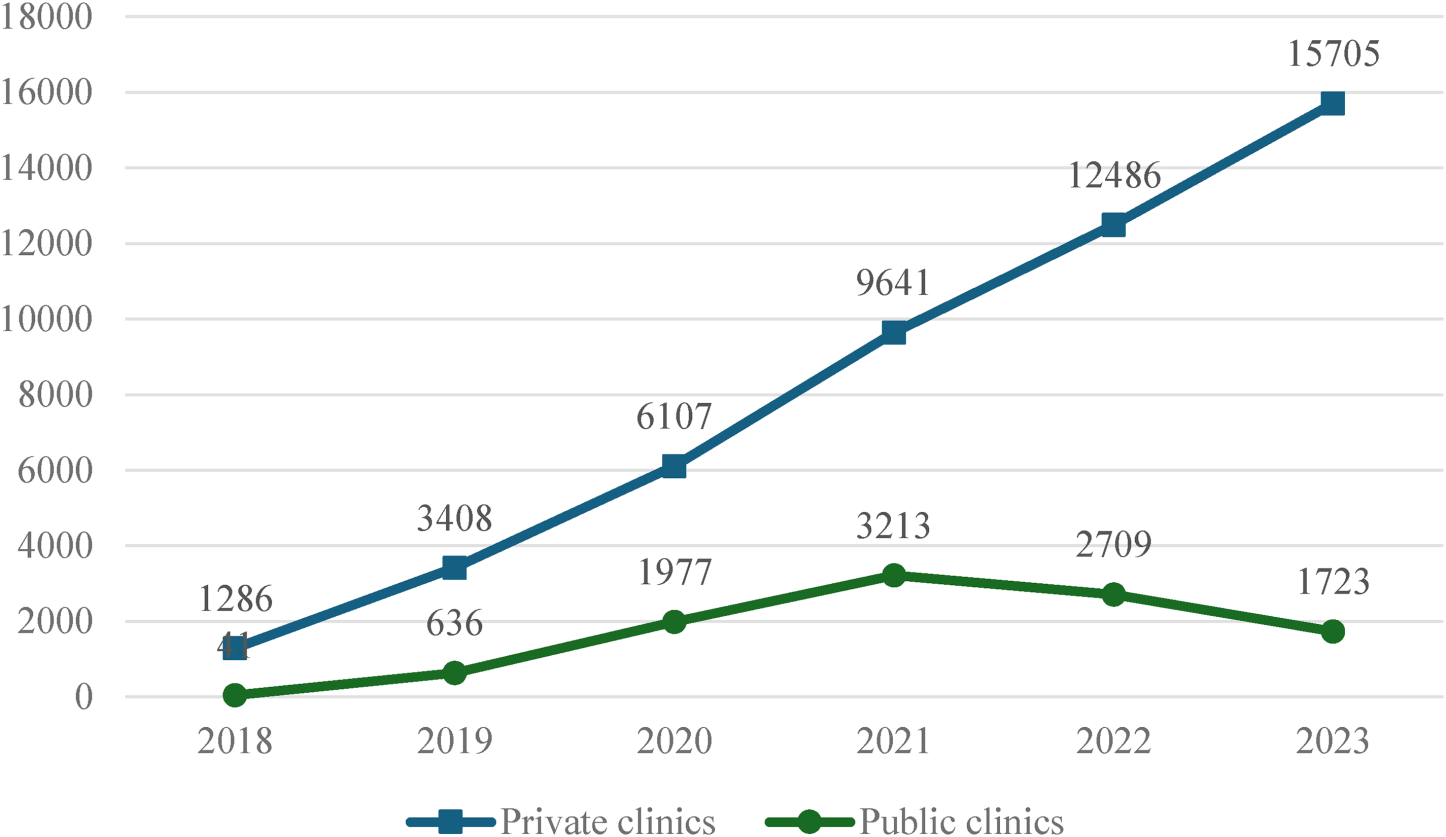
Annual PrEP uptake at private and public clinics, 2018–2023.

Over six years (2018–2023), we found that the number of people who received PrEP at least once in the last 12 months was consistently higher at private clinics compared to public clinics (Figure 2). Except for 2018, the number of PrEP clients at private clinics was three to nine times higher than public clinics (ratio: 31; 5;3;3;5;9, sequentially from 2018 to 2023). This reflects the substantial contribution of private sector service providers to PrEP uptake in the three project provinces.

### 3.3 PrEP persistence

**Figure 3:**
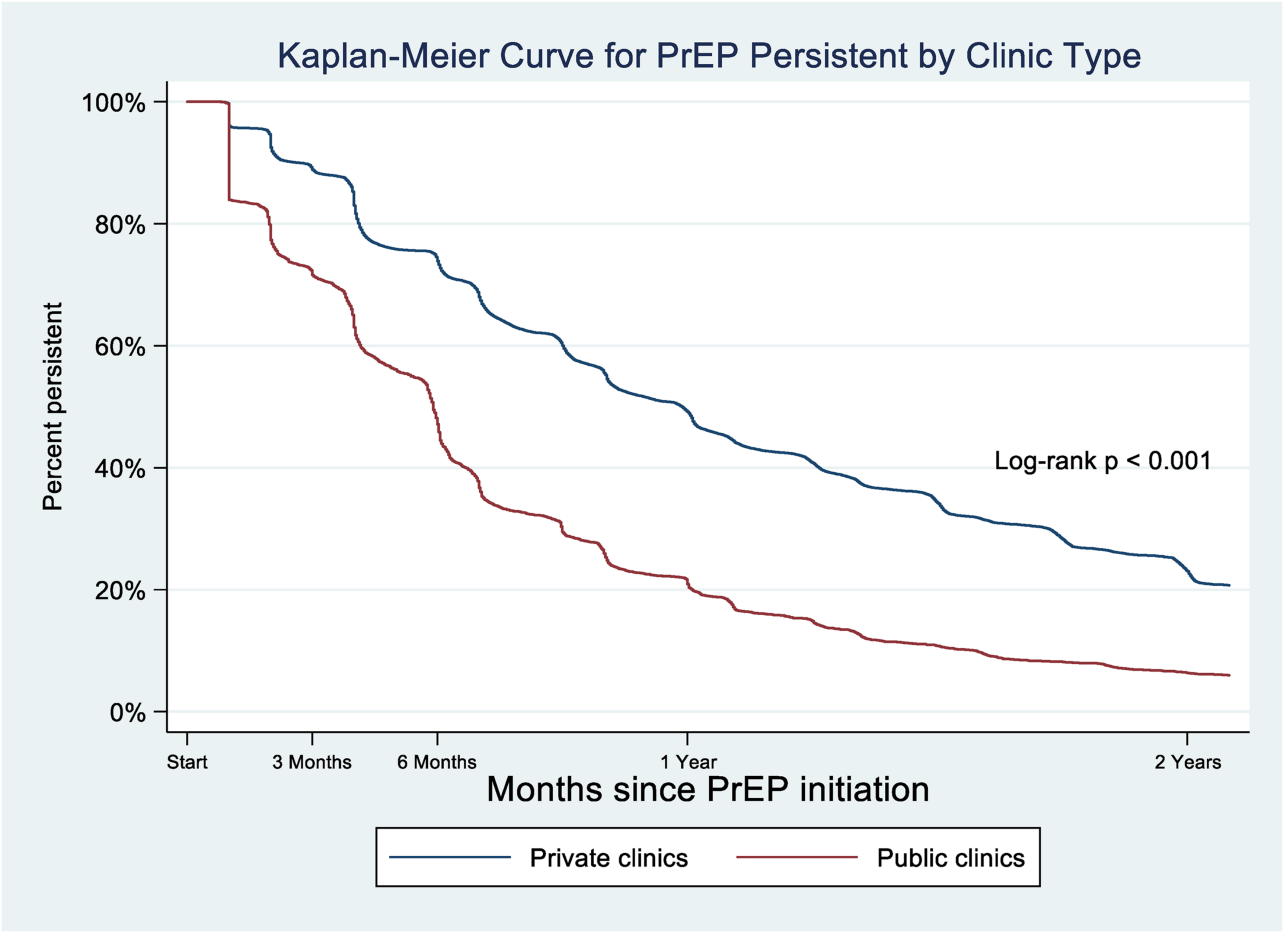
Kaplan–Meier curve of percentage of clients using PrEP who persisted with PrEP, by PrEP facility type (private vs. public clinics), 2018–2023.

Overall, the median persistence of PrEP users at private clinics was significantly longer than that at public clinics (268 vs. 148 days, respectively). The Kaplan-Meier curve illustrates the persistence of individuals on PrEP by facility type (private vs. public clinics) over a period of two years (Figure 3). We found that PrEP persistence among clients at private clinics was consistently higher than that at public clinics. After two years, PrEP persistence among clients at private clinics remained higher than that at public clinics. The steep decline trend of PrEP persistence at public clinics indicated a higher rate of PrEP discontinuation among clients at public clinics compared to private clinics (see Table 3 below). The Log-rank test confirmed a significant difference of PrEP persistence between private and public clinics (*p* < 0.001).

**Figure 4:**
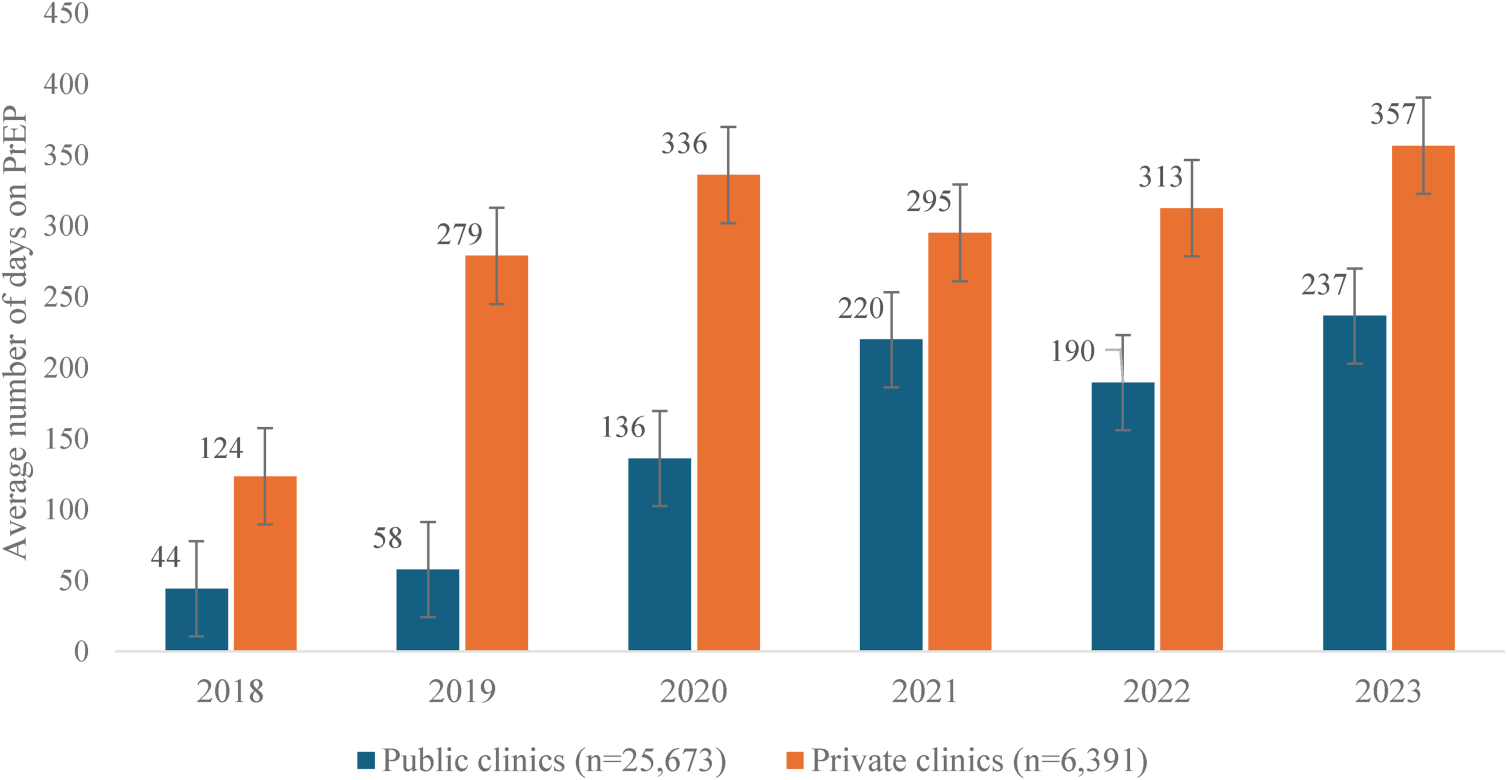
Average number of days on PrEP use among clients at private and public clinics, 2018–2023.

Figure 4 shows the trend of the average number of days on PrEP use among clients at private and public clinics over six years. The average number of days of PrEP use among clients at private clinics was consistently longer than that at public clinics throughout all six years though average number of days in the public sector has increased substantially since 2018 (ranging from 124 to 357 days with mean of 331 days vs. 44–237 days with mean of 204 days, respectively).

Overall, PrEP persistence rate at the three months (M3) at private clinics was consistently higher than at public clinics. Mixed-effects logistic regression analysis results indicated a significant association between PrEP persistence for at least three months and initiating PrEP at private clinics [adjusted odds ratio [aOR] = 4.28; 95% confidence interval [CI]: 2.96–6.19], opting for TelePrEP (aOR = 3.42; 95% CI: 2.12–5.53), or being a client at 20 years and older (aOR = 1.86; 95% CI: 1.62–2.13). In contrast, those who started PrEP through mobile PrEP services were less likely to be retained on PrEP for three months and longer (aOR = 0.57; 95% CI: 0.43–0.75) (Table 2).

**Table 2:** Factors associated with PrEP persistence for at least three months.

Variable	PrEP persistence ≥3 months	Mixed-effects logistic regression	
	*n* (%)	Adjusted OR (95% CI)	*p*-value
Age group (in years)			
15–19	1,352 (67.23%)	ref	
20–24	8,066 (80.5%)	1.86 (1.62–2.13)	<0.001***
25–49	16,027 (82.6%)	2.29 (2–2.63)	<0.001***
50+	353 (77.92%)	2.11 (1.57–2.84)	<0.001***
Key population group			
MSM	22,186 (81.6%)	ref	
TGW	907 (86.3%)	1.2 (0.98–1.49)	0.080
PWID	108 (49.77%)	3.01 (1.05–8.63)	0.040
FSW	523 (76.13%)	0.29 (0.2–0.4)	0.000
SDC	1,744 (76.56%)	0.84 (0.67–1.04)	0.110
Others	330 (71.12%)	0.87 (0.76–1.01)	0.060
PrEP facility type			
Public	4,398 (68.82%)	ref	
Private	21,400 (83.93%)	4.28 (2.96–6.19)	<0.001***
PrEP delivery model			
On site	25,283 (80.9%)	ref	
TelePrEP	242 (94.53%)	3.42 (2.12–5.53)	<0.001***
Mobile PrEP	273 (71.84%)	0.57 (0.43–0.75)	<0.001***
Fee-based PrEP			
No	22,043 (80.31%)	ref	
Yes	3,755 (84.57%)	0.75 (0.38–1.48)	0.400
PrEP client type			
New initiated client	23,045 (81.5%)	ref	
Re-initiated client	4,248 (72.6%)	0.91 (0.59–1.41)	0.68

Mixed-effects logistic regression test: repeated measurement by client unique ID code.

*** *p* < 0.001.

### 3.4 PrEP discontinuation and reinitiation

Notably, the proportion of clients who continued PrEP for three consecutive months and longer after initiation at private clinics was significantly higher than that at public clinics (85.3% vs. 70.9%, respectively, while the proportion of clients who continued PrEP for less than three months was lower than that at public clinics (14.7% vs. 29.1%, respectively; *p* < 0.001) (Table 3). Similarly, the proportion of clients who discontinued PrEP at more than three months after initiation at private clinics was significantly higher than that at public clinics (86% vs. 65.4%, respectively), while the proportion of clients who discontinued PrEP at less than three months after initiation was significantly lower than that at public clinics (14% vs. 34.6%, respectively; *p* < 0.001). The proportion of PrEP discontinuers who restarted PrEP at private clinics was significantly higher than that at public clinics (21.7% vs. 15.9%, respectively; *p* < 0.001).

**Table 3:** PrEP continuation, discontinuation and reinitiation at public and private clinics.

	Private Clinics	Public Clinics	*p* value
PrEP continuation	*n* = 25,673	*n* = 6,391	
3 + months	21,889 (85.3%)	4,530 (70.9%)	<0.001***
Less than 3 months	3,784 (14.7%)	1,861 (29.1%)	
PrEP discontinuation after initiation	*n* = 15,852	*n* = 4,434	
At more than 3 months	13,631 (86%)	2,900 (65.4%)	<0.001***
At less than 3 months	2,221 (14%)	1,534 (34.6%)	
PrEP reinitiation among PrEP discontinuers	3,440 (21.7%)	703 (15.9%)	<0.001***

Chi-square test.

*** *p* < 0.001.

**Figure 5:**
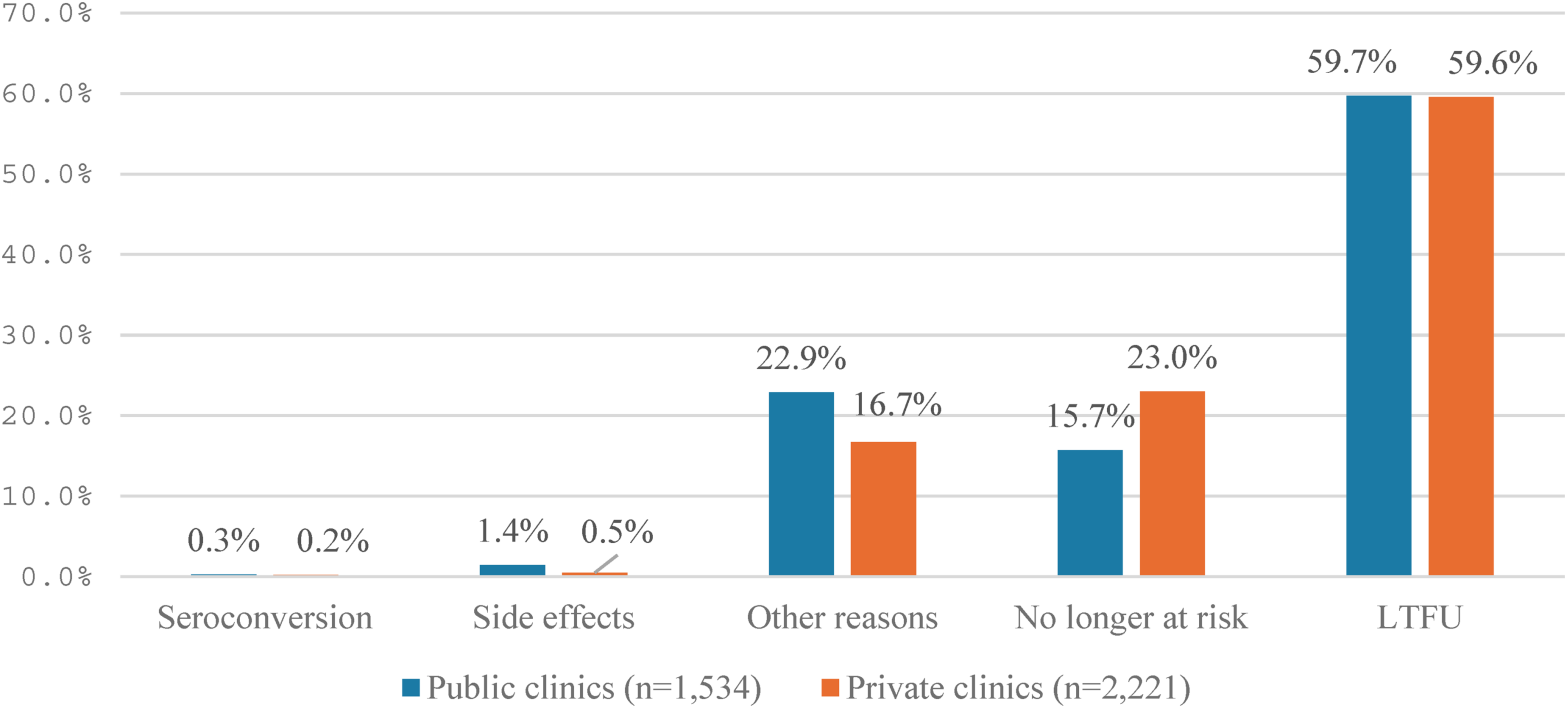
Reasons for discontinuation of PrEP use before three months after initiation at private and public clinics.

Reasons for discontinuation of PrEP use prior to three months after enrollment commonly reported by public and private clinics on the HMED system were lost to follow-up (LTFU), no longer at risk for HIV infection, and other personal reasons (e.g., changing living places and returning to hometown, particularly during COVID-19 in 2021), while side effects and HIV seroconversion were rarely reported (Figure 5). The reason “no longer at risk” was more likely to be reported by clients at private than at public clinics (23% vs. 15.7%, respectively). In comparison, the “other reasons” were more likely to be reported by clients in public than at private clinics (22.9% vs.16.7%, respectively).

**Figure 6:**
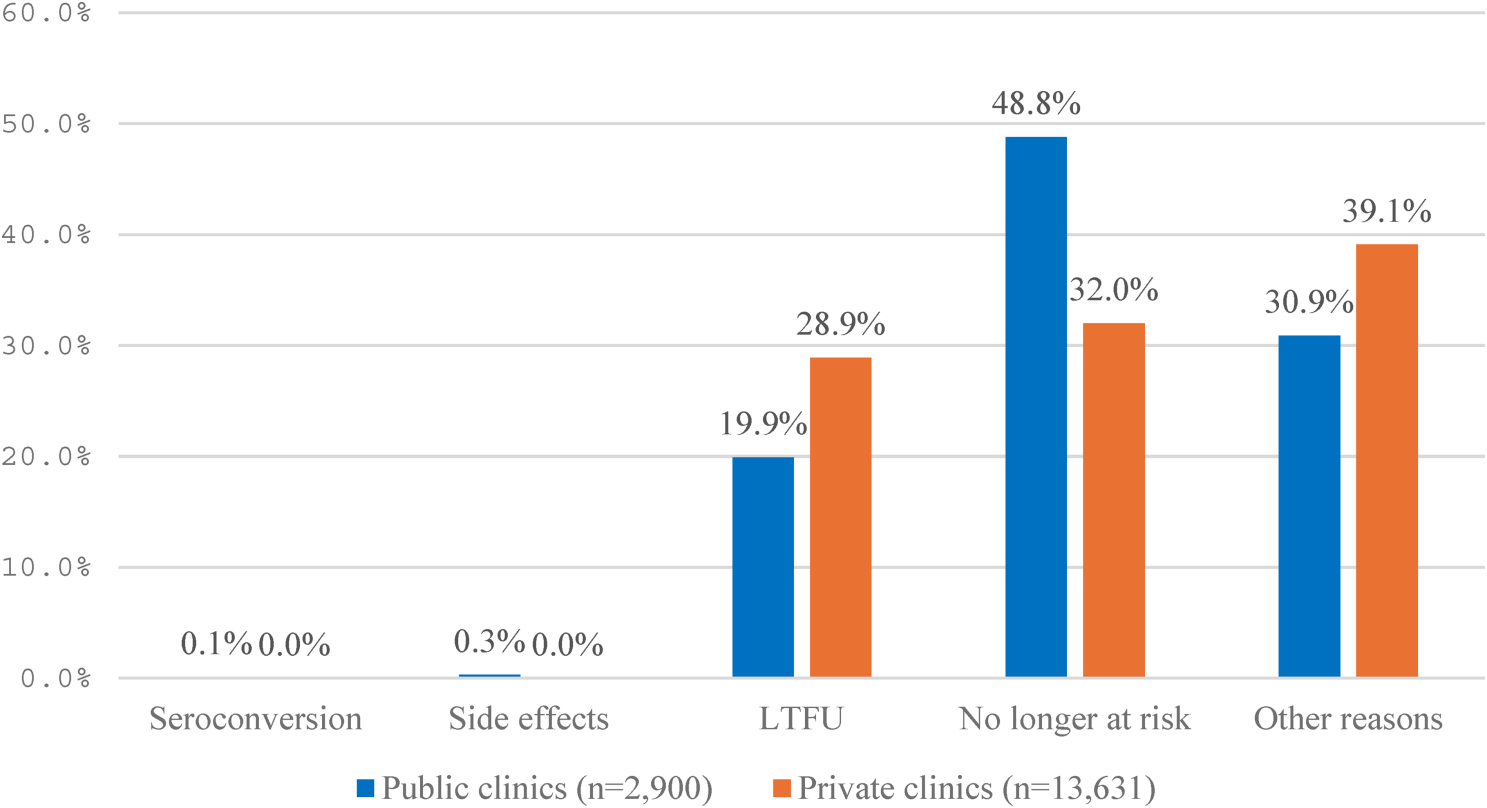
Reasons for discontinuation of PrEP use after 3+ months at private and public clinics.

Similarly, reasons for stopping PrEP use after 3 months and beyond were also LTFU, no longer at risk, and other reasons (e.g., changing living places and returning to hometown, particularly during COVID-19 in 2021). Clients at public clinics were more likely to report “no longer at risk” than at private clinics, while clients at private clinics were more likely to report “LTFU” and “other reasons” than at public clinics (28.9% vs. 19.9% and 39.1% vs. 30.9%, respectively) (Figure 6).

### 3.5 HIV seroconversion

**Figure 7:**
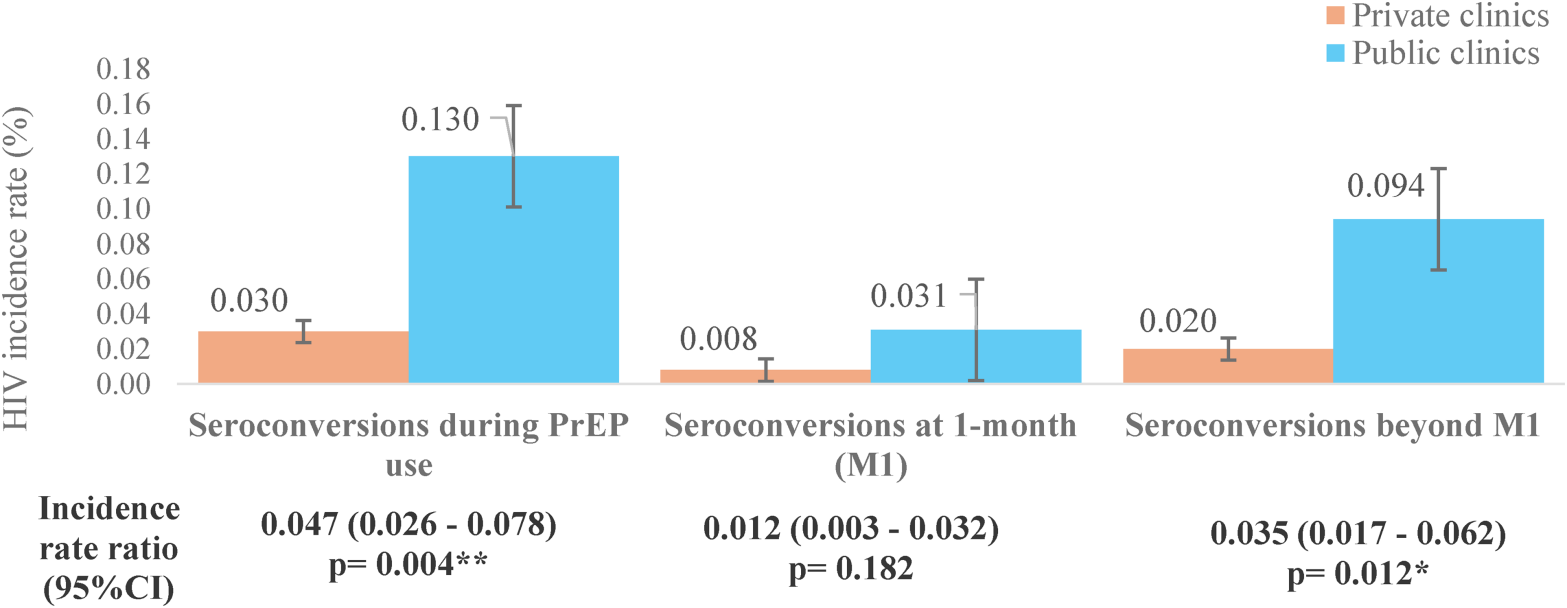
Observed HIV incidence rate among PrEP users at private and public clinics, 2018–2023. Fisher's exact test; *p < 0.05; **p < 0.01.

We identified 15 clients who HIV seroconversion among 31,887 daily oral PrEP users over six years. Overall, HIV seroconversion among PrEP users at both public and private clinics was extremely low (0.047 per 100 person-years-PY) (Figure 7). In other words, 95% of PrEP users would be protected from HIV infection during the time they were on PrEP use. Notably, the HIV seroconversion rate was significantly lower among PrEP users at private clinics compared to public clinics (0.03% vs. 0.13%, respectively; *p* < 0.01). This seroconversion rate was consistently lower at private clinics compared to public clinics after PrEP initiation at more than one month (0.02% vs. 0.094 per 100 PY, respectively; *p* < 0.05). There was no significant difference in seroconversion rate at the month one (M1) after initiation between private and public clinics.

## 4 Discussion

Private sector engagement in global health is a paradigm shift in the approach to development for greater impact and scale. PSE can result in greater scale, higher efficiency, accelerating innovation, creation of new markets, and enhanced sustainability of global health programs (20). Vietnam introduced the PSE approach in HIV response in 2014 in the context 80% of the national HIV programs were funded by external donors, and major donors had left or dramatically reduced the size and geographic scope of programs. USAID/PATH Healthy Markets (2014–2021) and USAID/PATH STEPS (2021–2026) were key partners of the Ministry of Health in implementing and scaling up PSE in new and innovative interventions, including PrEP. Although PSE in PrEP service delivery has been implemented across provinces and cities, supported by PEPFAR and GF, there was no published comprehensive analyses to demonstrate the effectiveness of the approach as part of a range of service options and its role in facilitating scale up of PrEP to achieve the HIV epidemic control in LMICs like Vietnam.

Our study assessed the effectiveness of engaging private sector health facilities in PrEP service delivery over six years in the national PrEP implementation program in Vietnam. We found that private sector PrEP service delivery played a critical role in scaling up PrEP services in the country. The private sector health facilities proved the ability to initiate PrEP uptake for a substantial number of people in need, successfully retain people on PrEP, and as a result, increase coverage of HIV prevention in Vietnam.

PrEP is a revolutionary HIV prevention tool, but high PrEP coverage must be achieved to have an impact on reductions in new HIV infection at the population level (1). PrEP uptake is a critical part of the PrEP cascade and is a key indicator to measure PrEP coverage (21–23). Given achieving the global PrEP targets by 2025 is far from being on track, the WHO strongly recommended the implementation of differentiated and simplified PrEP service delivery models to accelerate the attainment of the global targets (24). We found that the private sector PrEP service delivery model was successful in initiating PrEP for those in need. We posit that the reasons for success of private clinics in initiating and restarting PrEP may include offering client-centered care tailored to the needs of those that can benefit from PrEP, including MSM and TGW, greater perceived convenience, and providing more choice of PrEP service delivery options, such as on-site, mobile, or TelePrEP. In addition, PrEP services at private sector health facilities, particularly KP-led clinics are perceived as friendly, convenient, one-stop shopping experiences with flexible working hours. These advantages may make private clinics more attractive to MSM and TGW. In contrast, public clinics were able to recruit more serodiscordant couples, FSW, and PWID who are sexual partners of ART patients. Notably, KP-led private clinics that are KP-friendly and offer gender-affirming care counseling and testing were able to reach TGW, who are the most at-risk population, but hard to reach due to stigma and discrimination related to gender identity. This suggests that both public and private sector services are needed, and that private sector PrEP service delivery models plays a significant role in expanding PrEP coverage to key populations that are most in need of PrEP.

There has been concern over the persistence of PrEP use to ensure PrEP is highly effective in preventing HIV acquisition in any PrEP service delivery model. Good adherence to daily oral PrEP is associated with high prevention effectiveness, with at least four doses per week providing more than 90% efficacy for MSM, and poor adherence was associated with markedly reduced efficacy of PrEP (25–28). WHO guidelines suggest that PrEP can be stopped when persons are no longer at risk for HIV, and recommended client and provider decision-making regarding appropriate PrEP discontinuation (29). However, there are multiple patient-driven reasons for discontinuation, such as personal choice and preference, changes in life situation, and risk perceptions. We found an exceptionally high PrEP persistence among PrEP users at private sector PrEP service delivery sites compared to that at public sector PrEP sites. This finding is compatible with a study among young Thai men and transgender women that reported higher PrEP persistence (91.1%) at month 3 (30) but contrasts with studies among female sex workers (31) and adolescent girls and young women (32) in Africa that reported lower persistence at month 3 (33% and 34.9%). High PrEP persistence at the private clinics in this study may be related to the community engagement approach. MSM and TGW worked as service providers in diverse roles, such as clinicians, counselors, lab technicians, and pharmacists in KP-led private or non-KP-led private clinics. Private clinics also formed a strong partnership with community-based organizations (CBO) working as HIV case-finding partners, funded by PEPFAR and GF that supported PrEP referral, adherence counseling, and follow-up. In addition, private clinics maintained a reminder system via telephone calls or messages to clients before each scheduled visit and offered on-site testing and PrEP refills outside working hours. This indirectly reflects the quality of PrEP services provided by private service providers.

HIV seroconversion among PrEP users is a critical concern, as the goal of PrEP implementation is to prevent HIV acquisition at the individual level and drive HIV transmission toward elimination at the population level. Previous studies revealed a low rate of new HIV infections among people who took daily PrEP or lower HIV incidence among PrEP initiators compared to matched controls (30, 33, 34). These studies also observed that HIV seroconversions were mostly seen in those who stopped taking their pills. A multi-country demonstration study from West Africa showed that HIV seroconversions were higher for event-driven PrEP than daily PrEP (35), and it was significantly associated with persons who stopped PrEP. In general, we found a low HIV seroconversion rate among PrEP clients in our study, and the seroconversion rate was significantly lower among clients who opted for private sector PrEP service delivery compared to those who opted for public sector PrEP service delivery. This can be explained by the exceptionally high persistence rate among PrEP users at private clinics reported in the results section. In addition, private clinics maintained community peer counselors to support treatment adherence counseling to PrEP clients, using varied channels, such as telephone hotline, messaging via Zalo or Facebook, and face-to-face interactions during community outreach activities to remind clients prior to each scheduled visit and throughout their PrEP use course.

Limitations: Although the study's strength lies in its large sample size of PrEP clients and use of electronic medical records (EMR) from multiple sites in different geographic settings, the study has several limitations. First, the data is not representative of the national PrEP program. Second, the challenges inherent in collecting programmatic data within routine service delivery settings may occur missing or incomplete data. We proactively reviewed monthly progress indicator reports submitted by clinics and conducted routine DQA and CQI activities to improve the quality of data. Given the nature of retrospective programmatic data with basic indicators required by the national PrEP program, the ability to perform comprehensive data analysis to answer the research questions is limited, and the interpretation of results may be bias due to missing data or potential confounding factors. The retrospective study hinders the rigorous design to allow the comparison between public and private sector PrEP service delivery properly.

## 5 Conclusions

Engaging private sector service providers, such as private and KP-led clinics in PrEP service delivery is an effective approach to implement and scale-up PrEP in LMICs like Vietnam. Private sector service providers play a pivotal role in increasing PrEP uptake and expanding PrEP coverage. This study contributes to empirical evidence on the effectiveness of private sector engagement in PrEP service delivery that will reinforce ongoing efforts of the country to accelerate scaling up PrEP to achieve the ending AIDS goal by 2030. In line with this approach, the Ministry of Health approved the five-year national action plan on private sector engagement and investment in HIV response for the period 2021–2025, which is an excellent opportunity to increase scope and scale of private sector engagement in PrEP program throughout the country. This also includes engaging private pharmacies in PrEP service delivery to increase PrEP coverage and use, especially among populations not reached by clinic-based services. More research and programmatic evidence are needed to understand how best to implement and finance private sector engagement models to maximize impact particularly with the introduction of long-acting injectable PrEP products, such as cabotegravir and lenacapavir in LMIC settings.

## Data Availability

The original contributions presented in the study are included in the article/Supplementary Material, further inquiries can be directed to the corresponding author.

## Footnotes

1 Ministry of Health. *National PrEP Scale-up Plan for the Period 2021–2025 (Vietnamese Version)*. Hanoi (2020).

2 Ministry of Health. *National Guidelines on HIV/AIDS Care and Treatment*. Hanoi (2019).
